# Supplementary material for: Habitat selection of black grouse in an isolated population in northern Germany—the importance of mixing dry and wet habitats
Source: PeerJ. 2022 Oct 17;10:e14161. doi: 10.7717/peerj.14161 (PMC9583852; doi:10.7717/peerj.14161)
Supplement: Supplemental Information 1 [file peerj-10-14161-s001.docx]

| Grouped Habitat Types | Biotope Type Code | Habitats Directive Code | Habitats Directive Label | Biotope Types Label | Area [ha] |
| --- | --- | --- | --- | --- | --- |
| W | W |  |  | Forests (not further defined) | 6444.4 |
|  | WE | 91E1 | Alluvial forests with Alnus glutinosa and Fraxinus excelsior (Alno-Padion, Alnion incanae, Salicion albae) | Alder and ash forest of floodplains and spring areas | 28.8 |
|  | WA | 91E1 | Alluvial forests with Alnus glutinosa and Fraxinus excelsior (Alno-Padion, Alnion incanae, Salicion albae) | Alder-fen wood | 32.9 |
|  | WB | 91D0 | Bog woodland | Birch and pine fen wood | 239.3 |
|  | WN |  |  | Other swamp forest | 3.9 |
|  | WU | 91E1 | Alluvial forests with Alnus glutinosa and Fraxinus excelsior (Alno-Padion, Alnion incanae, Salicion albae) | Alder forest of drained sites | 18.6 |
|  | WV | 91D0 | Bog woodland | Birch and pine forest of drained bogs | 151.3 |
|  | WK | 9190 | Old acidophilous oak woods with Quercus robur on sandy plains | Pine forest of poor sandy soils | 932.5 |
|  | WP | 9120 | Atlantic acidophilous beech forests with Ilex and sometimes also Taxus in the shrublayer (Quercion robori-petraeae or Ilici-Fagenion) | Other pioneer and succession forest | 273.2 |
|  | WX |  |  | Other deciduous forest | 97.1 |
|  | WZ |  |  | Other coniferous forest | 5161.0 |
|  | WJ | 9110 | Luzulo-Fagetum beech forests | Young forest | 118.8 |
|  | WR |  |  | Structure-rich forest edge | 7.8 |
|  | UW | 2310 | Dry sand heaths with Calluna and Genista | Forest clearing corridor | 38.1 |
|  | WL | 9110 | Luzulo-Fagetum beech forests | Acidic beech forest | 329.6 |
|  | WQ | 9190 | Old acidophilous oak woods with Quercus robur on sandy plains | Acidic mixed oak forest | 609.7 |
|  | WC | 9160 | Sub-Atlantic and medio-European oak or oak-hornbeam forests of the Carpinion betuli | Mixed oak and hornbeam forest of nutrient-rich sites | 2.2 |
| Shr | HF |  |  | Other field hedge | 16.4 |
|  | HN |  |  | Near-natural field copse | 5.3 |
|  | HB | 9110 | Luzulo-Fagetum beech forests | Single tree/tree stand | 255.5 |
|  | HO |  |  | Scattered orchard | 4.4 |
|  | HP |  |  | Other woody plants/woody plantations | 1.2 |
|  | BW | 5130 | Juniperus communis formations on heaths or calcareous grasslands | Juniper scrub of soil acid sites (juniper heath) | 59.1 |
|  | BS |  |  | Soil-acid deciduous scrub | 6.0 |
|  | BN | 91D0 | Bog woodland | Bog and marsh scrub | 16.7 |
|  | BF |  |  | Other wetland scrub | 7.9 |
|  | BR |  |  | Ruderal shrubbery/other shrubbery | 5.7 |
| Bog | NS | 7140 | Transition mires and quaking bogs | Sour grass, rush and perennial reeds | 67.4 |
|  | NR |  |  | Land reed | 2.2 |
|  | NP | 7150 | Depressions on peat substrates of the Rhynchosporion | Other wet site with herbaceous pioneer vegetation | 1.8 |
|  | MH | 7110 | Active raised bogs | Near-natural raised bog of lowland | 9.6 |
|  | MW | 7140 | Transition mires and quaking bogs | Cotton grass stage of upland and transitional bogs | 27.8 |
|  | MG | 7120 | Degraded raised bogs still capable of natural regeneration | Bog heath stage of raised bogs | 23.4 |
|  | MP | 7120 | Degraded raised bogs still capable of natural regeneration | Pipegrass bog stage | 86.1 |
|  | MI | 7120 | Degraded raised bogs still capable of natural regeneration | Initial stage of wetted raised bogs | 1.5 |
|  | MZ | 4010 | Northern Atlantic wet heaths with Erica tetralix | Anemic and transitional bog heathland | 33.7 |
|  | MS | 7150 | Depressions on peat substrates of the Rhynchosporion | Bog stage with beaked reed vegetation | 1.8 |
| D | DO | 4030 | European dry heaths | Other open soil | 19.3 |
| HC | HC | 4030 | European dry heaths | Sand/silicate dwarf shrub heath | 4043.4 |
| Ng | RN | 6230 | Species-rich Nardus grasslands, on silicious substrates in mountain areas (and submountain areas in Continental Europe) | Nard grass meadow | 244.4 |
|  | RS | 2330 | Inland dunes with open Corynephorus and Agrostis grasslands | Sand dry grassland | 219.8 |
|  | RA | 4030 | European dry heaths | Species-poor heath or rough grassland stage | 328.0 |
| P | G |  |  | Greenland (not further defined) | 307.3 |
|  | GM | 6510 | Lowland hay meadows (Alopecurus pratensis, Sanguisorba officinalis) | Mesophilic grassland | 284.1 |
|  | GN |  |  | Sedge, rush or tall herb-rich wet meadow | 116.5 |
|  | GF | 3130 | Oligotrophic to mesotrophic standing waters with vegetation of the Littorelletea uniflorae and/or of the Isoeto-Nanojuncetea | Other species-rich wet grassland | 3.3 |
|  | GE |  |  | Species-poor extensive grassland | 213.3 |
|  | GI |  |  | Species-poor intensive grassland | 307.0 |
|  | GA |  |  | Grassland seeding | 40.5 |
|  | GW |  |  | Other pasture | 1.9 |
| Inf | GR |  |  | Shear grassland | 6.7 |
|  | HS |  |  | Woody vegetation of the settlement area | 4.5 |
|  | PH |  |  | Home garden | 11.2 |
|  | PA |  |  | Park area | 5.9 |
|  | O |  |  | Buildings, traffic and industrial areas | 365.6 |
|  | OV |  |  | Traffic area | 271.2 |
|  | OD |  |  | Village area/agricultural building | 24.1 |
|  | ON |  |  | Historical/other building complex | 3.2 |
